# The efficacy of peritubal analgesic infiltration in postoperative pain following percutaneous nephrolithotomy – A prospective randomized controlled study

**DOI:** 10.1590/S1677-5538.IBJU.2014.0471

**Published:** 2015

**Authors:** Bannakij Lojanapiwat, Tanarit Chureemas, Pruit Kittirattarakarn

**Affiliations:** 1 Division of Urology - Surgery 110 intravaroros Muang Chiangmai, Chiang Mai, Thailand; 2 Faculty of medicine - Surgery, Chiang Mai, Thailand

**Keywords:** Nephrostomy, Percutaneous, Postoperative Period

## Abstract

**Objective:**

To study the efficacy of peritubal infiltration in postoperative pain following percutaneous nephrolithotomy in general PCNL patients and PCNL patients with supracostal renal access.

**Patients and Methods:**

A total of 105 PCNL patients were randomized into two groups, 53 patients receiving peritubal analgesic infiltration (study group) and 52 patients as the control group. Of these patients, supracostal access was performed in 22 patients of study group and 23 patients of control group. The study group received peritubal injection with 10mL of bupivacain. Postoperative pain as the primary outcome was assessed by using visual analogue scale at 1, 4, 12, 24 and 48 hours postoperatively. The secondary outcomes were the total postoperative morphine usage in 24 hours and time of the first analgesic demand.

**Results:**

The average VAS pain at 1 and 4 hours after the operation in the study group were significant lower in the control group (P≤0.001 and 0.026). Doses of morphine usage for controlling postoperative pain and the first analgesic demand were significantly lower and longer in study group. Among patients submitted to supracostal access, the average VAS pain at 1 hour after operation in the study group was lower (P=0.018). Doses of morphine usage for controlling postoperative pain also was lower in the study group (P=0.012).

**Conclusion:**

The peritubal local anesthetic infiltration is effective in alleviating immediate postoperative pain after percutaneous nephrolithotomy even with supracostal access.

## INTRODUCTION

Traditionally, opioid analgesics such as meperidine and morphine are used in postoperative pain management. High doses of these drugs lead to higher rates of side effects including postoperative nausea and vomiting, drowsiness, respiratory depression, ileus, urinary retention and constipation (1, 3–6). Several techniques have been used to overcome these problems such as multimodal analgesic regimens, PCNL with small nephrostomy tube, tubeless PCNL, mini-PCNL, local analgesic infiltration and renal capsule analgesic infiltration (1, 2, 7–9). Another modality is peritubal local anesthetic infiltration which was developed under the rationale to relief the pain that might be originated in renal capsule after PCNL surgery (1, 10–12).

We studied the efficacy of peritubal infiltration of 0.25% bupivacaine in postoperative pain following percutaneous nephrolithotomy with percutaneous nephrostomy tube. We also studied the efficacy of this technique in patients with supracostal renal access.

## PATIENTS AND METHODS

### Patients

A total of 105 patients who underwent single tract PCNL with postoperative nephrostomy tube placement were recruited. The patients were randomized into two groups: 53 patients received peritubal analgesic infiltration (study group) and 52 patients were included in the control group. Twenty-two patients of study group and 23 patients of control group received supracostal access. Exclusion criteria included patients with a history of local analgesic allergy, patients who underwent a second nephroscopy, patients who required more than one puncture, and patients who had excessive intra-operative bleeding.

### Methods

After general anesthesia was administered, an open-end 6 F ureteral catheter was placed transurethrally into the ureter in supine position. Under fluoroscopic guidance in prone position, contrast media was injected via ureteral catheter. Renal access was created by the biplane technique of standard PCNL. For the supracostal access the needle puncture was performed through the diaphragm and retroperitoneum in full inspiration, whereas the needle was passed through the kidney during deep inspiration. After the tip of the needle was located in the collecting system, working and safety guide wires were inserted followed by tract dilatation with telescopic metal dilators sizes 8F to 30F with 30F Amplatz sheath. Stone was disintegrated with ultrasonic and/or pneumatic lithotripsy. The nephrostomy tube size 20F was routinely inserted in all cases.

**Figure 1:**
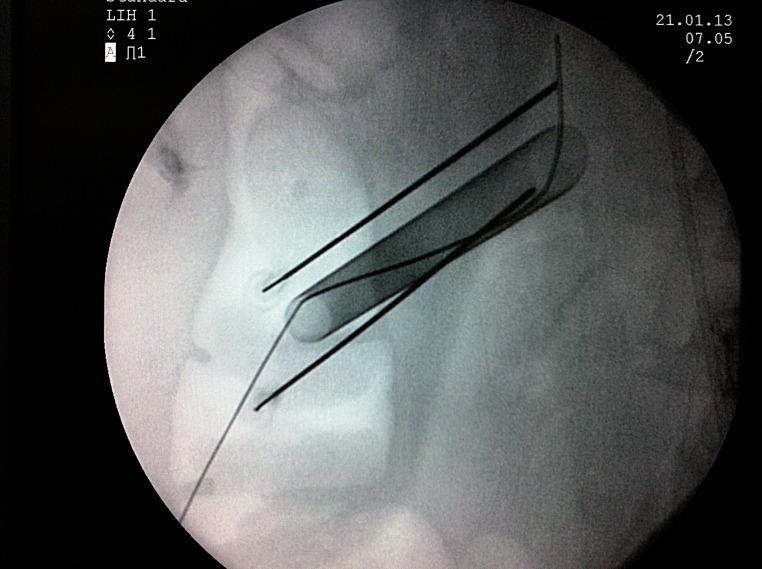
Intraoperative fluoroscopic view of peritubal injection.

In the patients of the study group, the 23-gauge, 90mm spinal needle was inserted up to the renal capsule under fluoroscopic guidance along the nephrostomy tube at 6 and 12 o'clock positions (cranial and caudal); then 0.25% bupivacaine was infiltrated into the nephrostomy tract, including renal capsule, muscle, subcutaneous tissue and skin, 10mL in each position (Figure-1). The control group did not receive any infiltration. Chest X-ray (CXR) and complete blood count were performed to evaluate blood loss and pulmonary complications.

Demographic and clinical characteristics of the patients were recorded at the time of enrollment. Postoperative pain as the primary outcome was assessed by an independent observer blinded to the infiltration using a 0–10 point visual analogue scale for pain (VAS pain) where 0 on the scale meant no pain and 10 meant very severe pain. VAS pain was recorded at 1, 4, 12, 24 and 48 hours postoperatively. The secondary outcomes were the total postoperative morphine usage in 24 hours, time of the first analgesic demand and adverse effects.

Statistical analysis was performed using SPSS® version 13. Continuous variables were compared using t-test for two independent samples. Categorical variables were compared using Chi-square analysis. P-value<0.05 was considered to be statistically significant.

All patients provided written informed consent. The ethical approval was obtained from the Institutional Review Board for human research project of Faculty of Medicine, Chiang Mai University.

## RESULTS

**Figure 2:**
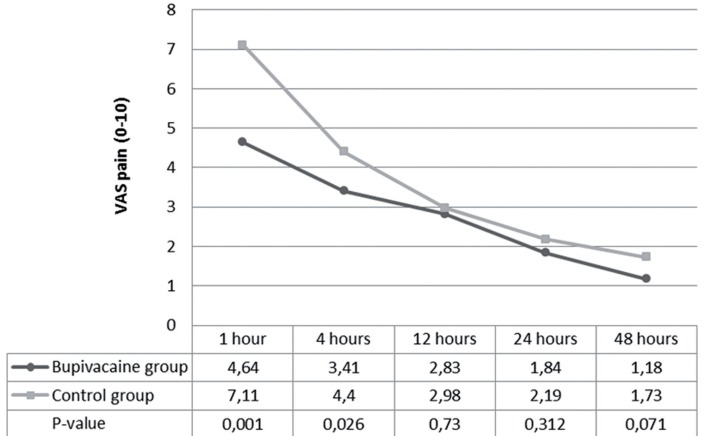
Visual Analog Score at postoperative times of 1, 4, 12, 24, 48 hours (total patients).

**Figure 3:**
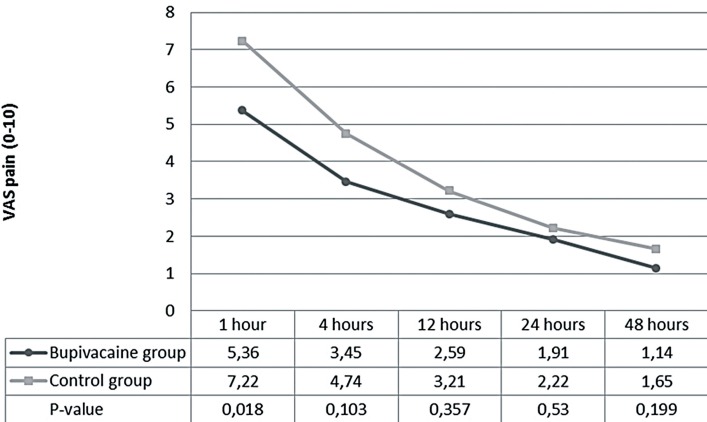
Visual Analog Score at postoperative times of 1, 4, 12, 24, 48 hours (supracostal patients).

Profiles of patients were not clinically significant different between the two groups (Table-1). Postoperative pain as the primary outcome evaluated by VAS is shown in Figure-2. The average VAS pain at 1 and 4 hours after the operation in the study group was 4.64±2.73 and 3.41±2.28 compared with 7.11±2.33 and 4.40±2.21 in the control group (P≤0.001 and 0.026), respectively. The postoperative VAS pain at 12, 24 and 48 hours were not significant different between both groups (Figure-3). Doses of morphine usage for controlling postoperative pain was 4.43±2.78mg in study group and 7.52±5.12mg in control group (P=0.002, Table-2). The first analgesic demand was longer in study group compared with the control group (97.00±87.74 min VS 55.10±60.50mg, P=0.007) (Table-2).

**Table 1:** profiles of patients (Total patients).

		Group I (Study group)	Group II P-value (Control group)	P-value
Patients		53	52	
Gender (M:F)		35:18	36:16	0.83
Age (years)		56.64+11.34	53.84±10.65	0.19
BMI		22.54±3.46	23.81±3.97	0.085
Stone size (cm)		4.00±1.83	4.05±1.88	0.845
**ASA status (n, %)**				
	ASA 1	20 (37.74)	18 (34.62)	0.94
	ASA 2	30 (56.60)	31 (59.62)	
	ASA 3	3 (5.66)	3 (5.77)	
Previous surgery (N, %)		10(19.87)	10 (19.23)	1.00
**Access site upper pole (N)**				
	Supracostal	22	23	0.847
	Subcostal	19	22	
	Middle	4	3	
	Lower	8	4	

**Table 2:** Clinical outcomes and complications (Total patients).

		Group I (Study group)	Group II (Control group)	P-Value
Stone Free (%)		38 (72%)	36 (69%)	0.84
Stone fragment≤4mm (%)		9 (17%)	10 (21%)	
Operative time (min)		90.09±28.05	86.15±27.45	0.46
Pulmonary complication		0	0	1.00
Morphine usage (mg)		4.43±2.78	7.52±5.12	0.002
First analgesic demand (min)		97.00±87.74	55.10±60.50	0.007
**Side effect (N, %)**				
	Nausea/vomiting	7 (13%)	13 (25%)	0.22

Supracostal access was performed in 55 patients, 22 patients in study group and 23 patients in control group. Profiles of patients are shown in Table-3. The average VAS pain at 1 hour after operation in the study group was 5.36±2.87 compared with 7.22±2.15 in the control group (P=0.018). The postoperative VAS pain at 4, 12, 24 and 48 hours were not significantly different between both groups. Doses of morphine usage for controlling postoperative pain was 4.92±2.96mg in study group and 8.81±6.36mg in control group (P=0.012, Table-4). The first analgesic demand was longer in study group compared with the control group, but was not significantly different (97.69±94.29min and 61.91±67.48min, P=0.165) (Table-4).

**Table 3:** Profiles of patients (supracostal access patients).

		Group I (Study group)	Group II (Control group)	P-value
Patients		22	23	
Gender (M:F)		17:5	17:6	1.00
Age (years)		54.18±9.33	53.69±9.32	0.86
BMI		23.50±2.75	24.89±3.60	0.16
Stone size (cm)		4.43±2.29	4.37±1.87	0.93
**ASA status (N, %)**				
	ASA 1	9 (40.9)	6 (26.09)	0.62
	ASA 2	11 (50.00)	14 (60.87)	
	ASA 3	2 (5.66)	3 (13.04)	
**Previous surgery**				
	(N, %)	6 (27.27)	5 (21.74)	0.74

**Table 4:** Clinical outcomes and complications (supracostal access patients).

		Group I (Study group)	Group II (Control group)	P-Value
Stone Free (%)		17 (78%)	18(79%)	0.87
Stone fragmen≤t4mm (%)		2 (10%)	3(13%)	
Operative time (min)		92.50±33.54	92.61±30.37	0.99
Pulmonary complication		0	0	1.00
Morphine usage (mg)		4.92±2.96	8.81±6.36	0.012
First time of analgesic demand (min)		97.69±94.29	61.91±30.37	0.165
**Side effect (N, %)**				
	Nausea/vomiting	2 (9.1%)	8 (34.79%)	0.03

## DISCUSSION

Postoperative pain is an important issue following the surgery. This affects the postoperative quality of life especially in recovery period with patient's anxiety and several negative aspects such as delayed mobilization, increased postoperative complications and prolonged hospitalization (3–6). Recently, several techniques have been developed for improvement of postoperative pain management due to better understanding of acute pain physiology, development of new analgesic agents, better analgesia delivery procedures and better local anesthetic infiltration techniques (10–15). Gender also affects the level of postoperative pain. Women have more pain sensitivity and therefore most women need more analgesic consumption than men (16, 17).

Percutaneous nephrolithotomy (PCNL) is accepted to be the minimally invasive procedure for large renal and ureteral calculi with less morbidity and mortality compared to open surgery. Even in minimally invasive nature, PCNL still causes significant postoperative pain especially in standard PCNL with nephrostomy tube. The purpose of nephrostomy tube placement following PCNL is for the tamponade of the bleeding along the tract, adequate drainage and maintenance of tract for a second nephroscope (8, 9). A significant number of PCNL patients have been distressed from postoperative pain mostly due to the presence of nephrostomy tube. Various techniques were reported to minimize postoperative pain following PCNL such as small bored nephrostomy tube, tubeless PCNL, lignocaine infiltration at renal capsule and peritubal infiltration (7–12).

Patients with small bored nephrostomy tube have less postoperative pain score and less narcotic requirement (2, 7). Tubeless PCNL is recommended in uncomplicated cases without increasing complication (8, 9). Techniques of small bored nephrostomy and tubeless PCNL have been shown to have the advantage of less postoperative pain, but these techniques are not recommended in patients with significant bleeding, significant extravasation and second nephroscope required (tubeless PCNL). As standard technique of PCNL, the placement of large nephrostomy tube follows completion of the procedure is recommended for general cases. Postoperative pain usually is caused by the dilatation of renal capsule and parenchyma of access tract with local inflammation reaction along the nephrostomy tube (10–12). Pain following PCNL that involved nephrostomy tube might originate from renal capsule, muscle, subcutaneous tissue and skin. Renal capsule and parenchyma are richly innervated of pain-conductive neurons; the pain is therefore not only at the skin (11).

Opioid analgesics are traditionally used for controlling postoperative pain, but these drugs usually have side effects. The usage of multimodal or a combination of lower doses of opioid analgesics with non-opioid analgesics could avoid these side effects. Several studies demonstrated the efficacy of acetaminophen with and without opioid in management of postoperative pain (3–6, 18, 19). Maghsoudi et al. reported the positive effect of intravenous paracetamol as part of multimodal analgesia regimen for postoperative pain management following PCNL. Fifty patients who received 1gram intravenous paracetamol had significantly less visual analog score at 6 and 24 hours postoperative period compared with patients that received placebo. The meperidine consumption was also lower in paracetamol group (54.40mg VS 77.60mg, P<0.001) (18).

The benefit of local anesthesia was demonstrated in previous studies of general surgery, gynecology and anesthesia such as cesarean sections, hysterectomy, thyroid surgery, mastectomy, total-hip arthroplasty and cervical spine surgery, where marcaine was used as anesthesia agent (20–23). From the previous studies, the maximal benefit of marcaine infiltration will be met if the infiltration is performed before the incision. Haleblian et al. studied the effect of local anesthetic (Marcaine®) infiltration at the incision wound (subcutaneous) of PCNL with 10 Fr. nephrostomy tube in 10 patients compared with 12 patients with saline infiltration. It was observed no significant differences between both groups in the aspect of pain scores and postoperative narcotic use. The sample size of the study was small and difficult to interpret, and marcaine was infiltrated subcutaneously, which was not adequate for the local pain control following this operation (11).

Jonnavithola et al. studied the randomized control of peritubal infiltration of bupivacaine of renal capsule and demonstrated the effectiveness of this technique. The technique consisted of the use of a 23 gauge spinal needle (10cm in length) along nephrostomy tube at 6 and 12 o'clock and each infiltrated 10mL of 0.25% bupivacaine. The pain free period and mean total consumption of tramadol following operation of controlled group and blocked group were 4.6±5.4 hours and 105±85 mg and 14.7±9.6 hours and 31±44 mg, respectively. The mean AUC-UAS was 39.2 hours in control group and 18.9 hours in infiltration group (12).

Ugras et al. demonstrated the positive effect on postoperative pain and ventilatory function following ropivacaine infiltration of skin, nephrostomy tract and renal puncture site in combination with parenteral analgesia (metamizol). The aim of the study was to evaluate visual analog score (VAS), peak expiratory flow rate (PEF) and blood gas analysis. The time of first analgesic demand, total analgesic need and VAS at 6 hours were significantly lower, and PEF at 2 and 6 hours were significantly higher in patients with combinded ropivacaine infiltration and parenteral analgesic. Combination treatment for postoperative pain control lead to better pain management, which resulted in better patient's ventilation (1).

Parikh et al. reported a prospective randomized study of the efficacy of 0.25% bupivacaine peritubal infiltration in 60 PCNL patients; 30 patients were included in the treated group (0.25% bupivacaine infiltration) and 30 in the controlled group (normal saline infiltration). Exclusive criteria of the study were multiple punctures, supracostal puncture, stone size larger than 2.5cm, duration of procedure more than 3 hours and excessive intraoperative bleeding. Visual analogue scale (VAS) and dynamic visual analogue scale (DVAS) were lower in bupivacaine injected patients in early and late postoperative times. Mean of first tramadol demand was significantly shorter in normal saline infiltration patients (1.96hours VS 4.4hours). Total tramadol consumption was higher in normal saline patients (276.8mg VS 119.3mg) (10).

Our patient recruitment criteria included all single tract PCNL patients with postoperative nephrostomy tube placement including supracostal puncture without considering operative time and stone size, which is different from previous studies. Our study confirms the benefit and safety of peritubal analgesic infiltration in controlling postoperative pain (lower VAS number), lower use of morphine and longer time of first analgesic requirement. These results were also observed in the subgroup analysis of supracostal access, which should have more pain after this operation.

## CONCLUSIONS

Peritubal local anesthetic infiltration with 0.25% bupivacaine resulted in beneficial effects in alleviating immediate postoperative pain after percutaneous nephrolithotomy even with supracostal access. This effect resulted in lower early postoperative pain (lower VAS score), lower number of morphine usage and longer time of first analgesic requirement.
